# Renal Complications After Pediatric Hematopoietic Stem Cell Transplantation: A Retrospective Cohort Study

**DOI:** 10.3390/jcm15207810

**Published:** 2026-10-10

**Authors:** Bengü Macit, Arzu Akyay, Yurday Öncül, Yılmaz Tabel, Ahmet Taner Elmas

**Affiliations:** 1 Department of Pediatric Hematology and Oncology, Malatya Training and Research Hospital, 44000 Malatya, Türkiye; 2 Division of Pediatric Hematology and Oncology, Department of Pediatrics, Faculty of Medicine, İnönü University, 44280 Malatya, Türkiye; 3 Division of Pediatric Nephrology, Department of Pediatrics, Faculty of Medicine, İnönü University, 44280 Malatya, Türkiye

**Keywords:** hematopoietic stem cell transplantation, acute kidney injury, chronic kidney disease, hypertension, pediatric patients

## Abstract

**Background/Objectives**: Kidney-related complications contribute substantially to morbidity in children undergoing hematopoietic stem cell transplantation (HSCT), although evidence in pediatric populations remains limited. This study aimed to evaluate the incidence of acute kidney injury (AKI), chronic kidney disease (CKD), and hypertension within the first two years following HSCT and to investigate factors associated with these complications. **Methods**: We retrospectively analyzed 70 pediatric patients who underwent HSCT at the İnönü University Pediatric Bone Marrow Transplantation Unit between November 2017 and December 2023; patients who died during follow-up were excluded. Renal function was assessed using serum creatinine, the glomerular filtration rate was measured by 24-h urine collection or estimated using the Schwartz formula, and renal imaging was performed. The Acute Kidney Injury Network criteria were used to classify AKI, whereas CKD was defined according to the Kidney Disease Outcomes Quality Initiative criteria. Associations with demographic characteristics, primary disease, conditioning regimen, nephrotoxic drug exposure, graft-versus-host disease, veno-occlusive disease, and infections were evaluated. **Results**: AKI occurred in 54.3% of patients during the first 100 days, comprising stage 1 in 30.0%, stage 2 in 17.1%, and stage 3 in 7.1%. Primary diagnosis, conditioning regimen, nephrotoxic drug use, and transplantation-related complications were not significantly associated with AKI. Hypertension developed in 32.9% of patients, with most cases occurring during the early period after transplantation. A significant association was observed between hypertension and myeloablative conditioning without total body irradiation (*p* = 0.014). CKD was present in 45.7% of evaluated patients at month 6 and 44.6% at month 12. Amphotericin B use was significantly associated with CKD at month 6 (*p* = 0.015). No patient developed end-stage kidney disease or required dialysis. **Conclusions**: Renal complications were common after pediatric HSCT. AKI occurred frequently during the first 100 days, while CKD remained prevalent at 6 and 12 months. Younger age and amphotericin B exposure were associated with CKD in this cohort. Early recognition of renal injury and long-term kidney follow-up are warranted after pediatric HSCT.

## 1. Introduction

Hematopoietic stem cell transplantation (HSCT) provides a potentially curative therapeutic option for a wide range of malignant and non-malignant hematological disorders, as well as metabolic diseases, immunodeficiency syndromes, and autoimmune diseases [1]. As survival following HSCT has improved, greater attention has been directed toward the assessment of transplantation-related complications. During the early period following transplantation, major complications include sepsis, acute graft-versus-host disease (GVHD), and veno-occlusive disease (VOD) [2]. Acute kidney injury (AKI) can be considered a result of these diseases or a complication on its own. In contrast, chronic kidney disease (CKD) may become apparent between 3 and 12 months following transplantation [3]. Several factors may contribute to the development of CKD, including TBI, nephrotoxic antibiotics, infections, cyclosporine A, amphotericin B, chronic GVHD, and the cumulative nephrotoxic effects of chemotherapeutic agents [4]. Underlying diagnoses and treatments administered before HSCT may adversely affect renal function. Renal function may be influenced differently by transplantation type (allogeneic or autologous), donor type (related or unrelated), the degree of donor–recipient HLA matching (fully matched or mismatched), and the conditioning regimen (myeloablative or non-myeloablative). Given the limited evidence regarding nephrological complications in pediatric patients after HSCT, this study aimed to evaluate acute and chronic nephrological problems occurring within two years following transplantation and to investigate their potential causes.

## 2. Materials and Methods

### 2.1. Study Design and Population

The study included 70 patients who underwent HSCT at the İnönü University Turgut Özal Medical Centre Paediatric Bone Marrow Transplantation Unit from November 2017 to December 2023. Patients who died were excluded from the study. Ethical approval for the study was granted by the İnönü University Health Sciences Scientific Research Ethics Committee (Decision No. 2025/7033; approval date: 4 February 2025). Written consent for the use of clinical data for scientific purposes and publication was obtained from the parents or legal guardians of all patients in accordance with institutional procedures.

### 2.2. HSCT Procedure

Based on their diagnoses, the patients received treatment regimens as myeloablative treatment with TBI, myeloablative treatment without TBI or non-myeloablative treatment. Cyclosporine A (CsA) was initially administered parenterally at 3 mg/kg/day from day 1 and was switched to oral administration 20–30 days later. From day +60, the dose was tapered by 10% each month and discontinued at month +6 in the absence of GVHD. The CsA dose was adjusted to maintain blood levels between 150 and 200 ng/mL, and CsA concentrations were determined using a nonspecific fluorescence polarization immunoassay (TDx; Abbott Laboratories, Chicago, IL, USA).

All patients were managed in laminar airflow units and received prophylactic fluconazole (5 mg/kg/day) and acyclovir (1500 mg/m^2^/day), starting on day −1 and continuing until days +75 and +180 after transplantation, respectively. Cotrimoxazole (150 mg/m^2^/day) was administered until day −1 and resumed after neutrophil engraftment. Ciprofloxacin (20 mg/kg/day) was administered for antibacterial prophylaxis. In neutropenic patients, cefepime-sulbactam (90 mg/kg/day), amikacin (15 mg/kg/day), and teicoplanin (10 mg/kg/day) were initiated when the first recorded temperature was ≥38 °C or when a temperature of ≥37.5 °C persisted for more than one hour. Liposomal amphotericin B (3 mg/kg/day) was added if fever persisted for 72–96 h.

### 2.3. Renal Assessment and Definitions

The mean serum creatinine level measured at least twice before the conditioning regimen was accepted as the baseline basal creatinine level. Serum creatinine levels were obtained daily from HSCT to discharge. The highest serum creatinine level and the corresponding measurement day were recorded during the first 100 days after transplantation. Following discharge, measurements were performed monthly during the first year and every two months thereafter. Creatinine concentrations in serum and urine were measured using the Jaffe method.

The glomerular filtration rate (GFR) was determined using 24-h urine collection. GFR was derived using the following equation: GFR (mL/min/1.73 m^2^) = urine volume (mL/min) × urine creatinine (mg/dL) × 1.73 m^2^/serum creatinine (mg/dL). GFR measurements were obtained before the conditioning regimen, at discharge, and at 3, 6, and 12 months after transplantation. AKI was evaluated on the basis of serum creatinine levels and staged using the Acute Kidney Injury Network (AKIN) classification system [5]. The peak creatinine value and the corresponding day of measurement were recorded. Stage 1 corresponded to an increase in serum creatinine to 1.5–1.99 times the baseline value, stage 2 to an increase of ≥2 but <3 times the baseline value, and stage 3 to an increase of ≥3 times the baseline value. CKD was defined according to the criteria described in reference [6], based on kidney abnormalities persisting for more than 3 months. In patients aged ≥ 2 years, CKD was defined as a GFR < 60 mL/min/1.73 m^2^ for more than 3 months or, in patients with a GFR ≥ 60 mL/min/1.73 m^2^, by the presence of markers of kidney damage. GFR categories were defined as follows: stage 1, GFR ≥ 90 mL/min/1.73 m^2^ with evidence of kidney damage; stage 2, GFR 60–89 mL/min/1.73 m^2^ with evidence of kidney damage; stage 3a, GFR 45–59 mL/min/1.73 m^2^; stage 3b, GFR 30–44 mL/min/1.73 m^2^; stage 4, GFR 15–29 mL/min/1.73 m^2^; and stage 5, GFR < 15 mL/min/1.73 m^2^ [6]. For children younger than 2 years, renal function was interpreted using age-specific GFR reference values. CKD classification at months 6 and 12 was based on persistent rather than isolated abnormalities; a single transient reduction in GFR was not considered sufficient for the diagnosis of CKD. Proteinuria and albuminuria were not systematically assessed and were therefore not included as separate variables in the analysis. Estimated GFR was calculated in younger patients who had difficulty in collecting urine by using the Schwartz equation (Schwartz equation: 0.413 × [height (cm)/Plasma Creatinine (mg/dL]).

Dimercaptosuccinic acid (DMSA), diethylenetriaminepentaacetic acid (DTPA), and renal ultrasonography (USG) were performed before transplantation to assess renal function and structural abnormalities; abnormalities detected by these assessments were considered markers of kidney damage for CKD classification.

### 2.4. Clinical Variables and Outcomes

Data on major clinical problems, hepatic and renal biochemical parameters, and exposure to potentially nephrotoxic drugs (CsA, aminoglycosides, vancomycin, and amphotericin B) were retrospectively collected for the two-year periods before and after HSCT. CsA toxicity was considered present when serum creatinine increased by ≥50% in the absence of another identifiable cause of AKI and renal function improved following CsA dose reduction [7]. The diagnosis of GVHD was based on the Mount Sinai AGVHD International Consortium (MAGIC) criteria, whereas VOD was diagnosed using the Seattle criteria [8,9].

### 2.5. Statistical Analysis

Data were analyzed using IBM SPSS Statistics version 26.0 (IBM Corp., Armonk, NY, USA). Categorical data were summarized as numbers and percentages, whereas continuous data were expressed as mean ± standard deviation or median (minimum–maximum), as appropriate. Comparisons of categorical variables were performed using the chi-square test. When two independent groups were compared, continuous variables were analyzed with the Mann–Whitney U test if they were non-normally distributed and with Student’s *t*-test if they were normally distributed. For analyses involving three or more independent groups, the Kruskal–Wallis test was applied to non-normally distributed variables, whereas one-way analysis of variance (ANOVA) was applied to normally distributed variables. Tukey’s test was used for post hoc comparisons. Differences in AKI development according to primary disease were assessed using the log-rank test. Statistical significance was defined as a two-sided *p* value < 0.05.

## 3. Results

Of the patients, 40% (*n* = 28) were diagnosed with thalassemia major (TM), 21.4% (*n* = 15) were diagnosed with malignant diseases (hematologic malignancies, NHL, HL, Ewing sarcoma), 17.1% (*n* = 12) were diagnosed with Fanconi aplastic anaemia (FAA) and 21.4% (*n* = 15) were diagnosed with other diseases (acquired aplastic anaemia, PNH, myelodysplastic syndrome, adrenoleukodystrophy, immunodeficiency). Descriptive characteristics of the patients are shown in Table 1. Regarding transplantation characteristics, 2 patients underwent autologous HSCT, 61 underwent allogeneic HSCT from related donors, and 7 underwent allogeneic HSCT from unrelated donors. Among the related donors, 58 were siblings and 3 were other family members. Bone marrow was used as the stem-cell source in 45 patients and peripheral blood in 25 patients. Conditioning consisted of myeloablative regimens with TBI in 9 patients, myeloablative regimens without TBI in 42 patients, and non-myeloablative regimens in 19 patients.

Table 2 shows the pre-HSCT nephrological evaluations of patients and their distribution by diagnoses. No significant difference was found in mean basal creatinine levels among the diagnostic groups (*p* = 0.115). The mean basal GFR level was 107.4 ± 44.8. The GFR level was 73.4 ± 34.1 in patients with FAA, 119.8 ± 40.5 in patients diagnosed with TM, 110.4 ± 57.4 in patients diagnosed with malignancies and 110.3 ± 29.1 in patients with other diagnoses.

**Figure 1:**
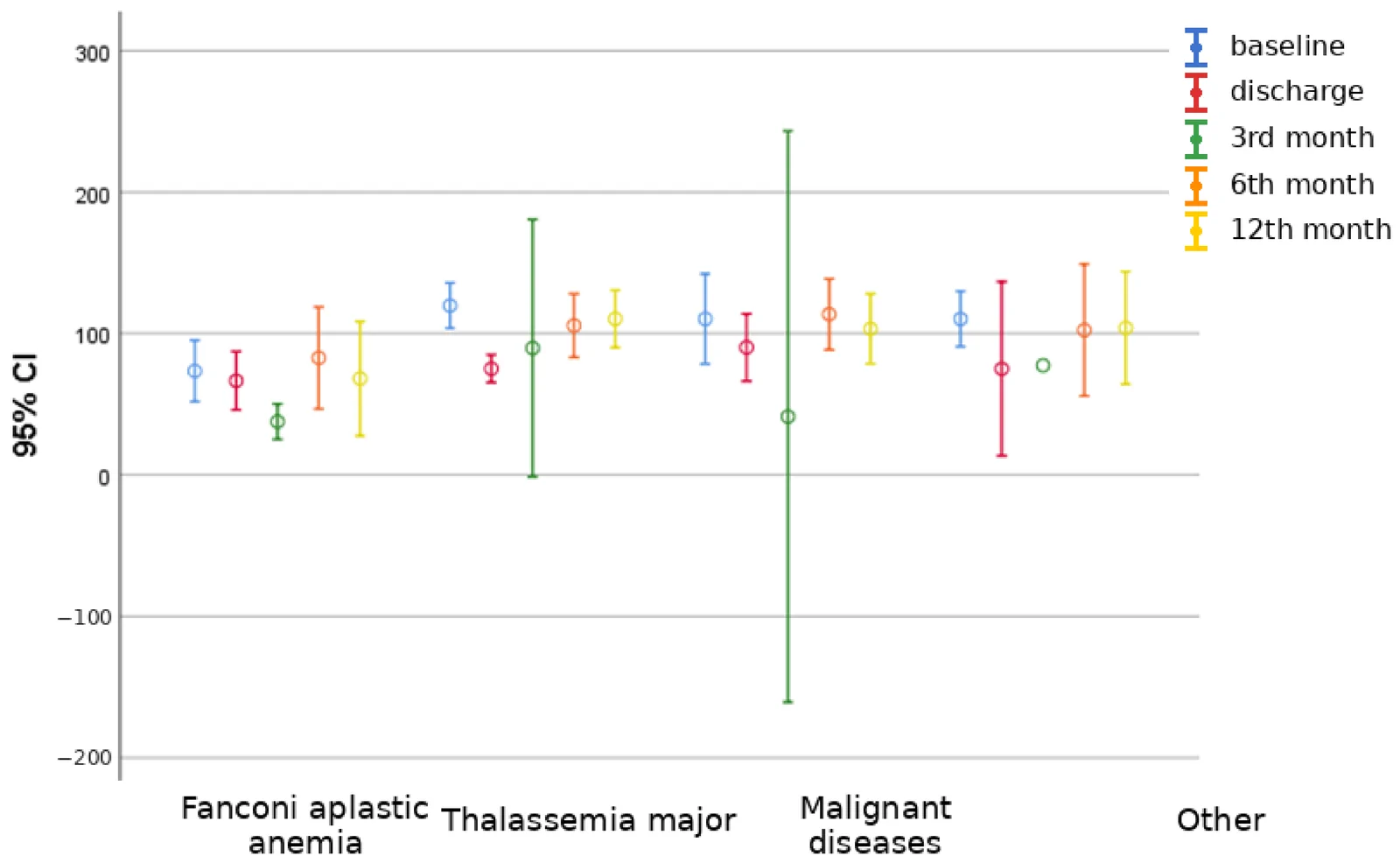
Mean GFR values in terms of diagnoses and time.

The changes in the mean and 95% confidence intervals of GFR values before HSCT and at discharge, month 3, month 6 and month 12 according to diagnoses are shown in Figure 1. Basal GFR values of the patients with FAA were significantly lower than those of the patients with TM (*p* = 0.010). No significant differences were found among the diagnostic groups in terms of the other GFR values (*p* > 0.05).

When creatinine levels of patients were evaluated in terms of AKIN classification, AKI developed in 54.3% (*n* = 38) of the patients within the first 100 days, according to the highest creatinine level. Changes in AKI status over time in terms of diagnoses are shown in Table 3. No cases of Stage 2 or Stage 3 AKI were found at months 6, 12 or 24.

**Figure 2:**
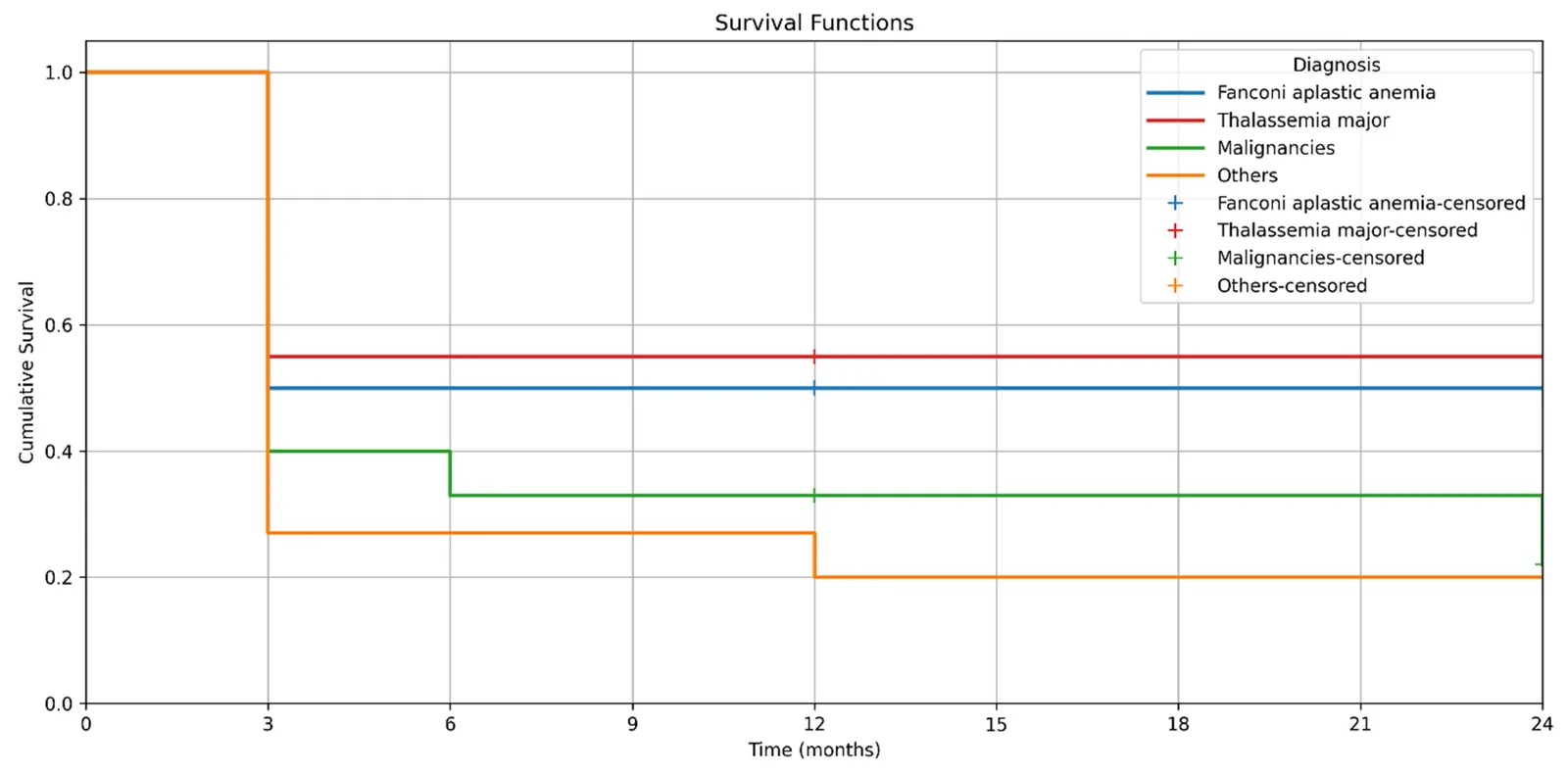
Patients’ AKI development status at any time up to the relevant period after HSCT, according to their diagnoses (Logrank test *p* = 0.104). The plus (+) symbols indicate censored observations.

No significant difference was found between patients’ primary diagnoses after HSCT and occurrence of AKI (*p* = 0.104) (Figure 2).

The comparison of patient characteristics according to AKI classification based on the highest creatinine value within 100 days is shown in Table 4, and no significant difference was found (*p* > 0.05).

No significant difference was found in the development of HT after HSCT, complication status, CMV infection, medication use, or conditioning regimen in terms of AKI development (*p* > 0.05) (Table S1).

HT developed in 32.9% (*n* = 23) of the patients after HSCT. The median time to HT development was 4 days (range, 1–28 days), and the median time to recovery was 18 days (range, 5–365 days), indicating that HT occurred predominantly in the early post-transplant period and resolved within 1 year in all affected patients. Primary diagnoses, medication use, USG findings, CMV infection and complications of the patients are shown in Table 5, and no significant difference was found between these and HT (*p* > 0.05).

No cases of HT developed after HSCT in patients who received myeloablative treatment with TBI, while HT developed in 45.2% of patients who received myeloablative treatment without TBI (*n* = 19) and in 21.1% of patients who received non-myeloablative treatment (*n* = 4), and a significant difference was found between them (*p* = 0.014). In post hoc evaluation, the incidence of HT was significantly higher in patients who received myeloablative therapy without TBI compared to those who received myeloablative therapy with TBI (*p* = 0.033). The creatinine and GFR values of patients who developed HT after HSCT and those who did not are shown in the table. Patients who did not develop HT had significantly higher creatinine levels at 12 months compared to those who developed HT (*p* = 0.019). Patients who did not develop HT had significantly higher GFR values at 6 months compared to those who developed HT (*p* = 0.029) (Table 5).

CKD status could be evaluated in 46 of the 70 patients at month 6 and in 56 patients at month 12 based on the availability of renal assessment data at the respective time points. Accordingly, the CKD frequencies reported at months 6 and 12 represent the proportions among patients with available renal assessments at each time point and should not be interpreted as prevalence estimates for the entire original cohort (Table 6).

Table S2 shows the comparison of CKD status at months 6 and 12 according to patient characteristics. The incidence of CKD was significantly higher in patients who used amphotericin B (*p* = 0.015). No significant difference was found in the incidence of CKD in terms of other characteristics (*p* > 0.05).

The ages of patients with CKD at both 6 months and 12 months were significantly lower than those without CKD (*p* < 0.05). No significant difference was found in creatinine levels between patients with and without CKD at both the 6th and 12th months (*p* > 0.05) (Table 7).

None of the patients followed developed acute or chronic renal failure. No patients needed dialysis.

## 4. Discussion

Acute and chronic kidney problems are the most important complications of HSCT. There are a limited number of studies on this topic in children. The present study has important findings regarding post-HSCT kidney problems. In the evaluation of our patients according to AKIN criteria, AKI was found to develop in 54.3% within the first 100 days after HSCT. In a study conducted by Koh et al., who used AKIN criteria to evaluate AKI, the incidence of AKI was found to be 68.2% in the first 100 days after the transplantation, while İleri et al. found the incidence of AKI to be 42% according to AKIN criteria. The incidence of AKI was similar to the literature in the present study [2,10].

Early and accurate diagnosis of AKI may improve clinical outcomes; however, the lack of standardized diagnostic criteria and classification remains a challenge. Various definitions and classification systems for AKI have been applied across studies. In a study conducted by Kizilbas et al., who used pRIFLE criteria in the evaluation of AKI, the incidence of AKI within the first 100 days after transplantation was reported as 84% [11]. Another study using the Bearman criteria, which define stage 1 AKI as an increase in serum creatinine of up to twofold from baseline, reported stage 1 AKI in 92–93% of patients [7,12]. In a study conducted by Zappitelli et al. on paediatric patients, it was reported that changes in GFR resulted in a higher AKI prevalence compared to changes in serum creatinine [13]. No significant correlation was found in the present study between AKI and GFR level. The use of different criteria across studies highlights the need for a standardized definition of AKI for both clinical practice and research. The AKIN criteria provide a standardized framework for the diagnosis and classification of AKI [5]. According to the AKIN criteria, stage 1 AKI is defined as a 1.5- to 1.99-fold increase in serum creatinine from baseline. Based on this definition, 21 (30%) of our patients were classified as having stage 1 AKI. This definition may help identify patients with mild impairment of kidney function.

In an adult study, dialysis-requiring AKI was reported to be rare following non-myeloablative HSCT [14]. In another study, a myeloablative treatment regimen was reported to be a risk factor for AKI [10]. In a study conducted by Frisk et al., TBI use was associated with AKI [15]. In the present study, no significant correlation was found between conditioning regimen and AKI. No relationship was found between sex and AKI development in the present study. The heterogeneous underlying diagnoses and transplantation characteristics may have limited the ability to identify demographic associations with AKI.

In malignant diseases, while the risk of AKI development is expected to be higher than in other diseases since the risk of renal involvement is higher due to both the intensity of the conditioning regimen and the chemotherapy received for the primary disease, in the present study and in the study conducted by Matsuake et al. [1], no significant difference was found between primary diagnoses and the risk of developing AKI.

In the present study, five patients had CsA toxicity and all developed AKI. Renal function returned to normal after CsA dose reduction. Nephrotoxic medication exposure is recognized as an important contributor to kidney injury after pediatric HCT [16]. In the present study, no significant association was found between amphotericin B exposure and AKI; however, CKD development at month 6 was significantly more frequent among patients who received amphotericin B.

In the present study, while no significant correlation was found between AKI and aGVHD, AKI developed in 87.5% of the patients who developed aGVHD. In a study conducted by Gurbanov et al., similar to the present study, no significant correlation was found between AKI and VOD [17]. There are studies that showed a significant correlation between VOD and AKI development [2]. This situation may be related to the low number of patients developing VOD after HSCT. AKI developed in 75% of our patients who used aminoglycosides. As stated in the literature, this situation may be related to the nephrotoxic effect of aminoglycoside use [18].

An important complication related to HSCT is hypertension (HT). Data on HT after pediatric HSCT remain limited. HT was observed in 32.9% of our patients, predominantly in the early post-transplant period, and resolved within 1 year at the latest. In the present study, HT was more frequent among patients who received myeloablative conditioning without TBI than among those who received myeloablative conditioning with TBI or non-myeloablative conditioning. However, only nine patients received myeloablative conditioning with TBI, and the absence of HT in this small subgroup should not be interpreted as evidence of a protective effect of TBI. This unadjusted association should be interpreted cautiously because the conditioning regimen may be related to underlying diagnosis, age, transplantation characteristics, baseline renal function, and other clinical factors, resulting in potential confounding. Therefore, no causal relationship between conditioning regimen and post-transplant HT can be inferred from these findings. Patients who did not develop HT had significantly higher GFR values at 6 months than those who developed HT. Hypertension and other kidney complications have also been reported during long-term follow-up of pediatric HSCT recipients [17].

CKD is an important late complication of HSCT, although reported frequencies vary substantially according to patient characteristics, follow-up duration, and the definitions used. A recent systematic review and meta-analysis of 54 pediatric HSCT studies reported a pooled proportion of late kidney outcomes of 15% and highlighted substantial heterogeneity in kidney outcome definitions [19]. In the present study, CKD was identified in 45.7% of evaluated patients at month 6 and 44.6% at month 12. These relatively high rates should therefore be interpreted in the context of differences in CKD definitions, patient populations, and follow-up assessments. None of our patients required dialysis. No relationship was found between AKI and CKD development in the present study, although acute renal insufficiency has been identified as a predictor of later CKD after pediatric HCT [20].

In a study conducted by Berg et al. [21], long-term kidney function was evaluated in children after HSCT performed due to acute leukaemia and severe aplastic anaemia, and a significant decrease was found in mean GFR (inulin clearance) one year after HSCT, with GFR continuing to be significantly lower than in controls during five years of follow-up. In a study conducted by Lugthart et al. [22] on 216 pediatric patients, hematological malignancy as the HSCT indication was an independent risk factor for CKD. However, in the present study, GFR was lower than in other diagnostic groups in patients with FAA both before and after transplantation. This may be associated with structural kidney anomalies related to the primary disease in patients with FAA.

Frisk et al. [15] reported TBI as a cause of CKD. However, in the present study, no significant unadjusted association was observed between either TBI or aGVHD and CKD; similarly, Selistre et al. [23] did not report a significant association. At month 6, CKD was more frequent among patients exposed to amphotericin B; however, this finding was based on a very small exposed subgroup (5 of 6 patients) and should be interpreted cautiously. This observed unadjusted association does not establish a causal relationship, and confounding by indication cannot be excluded. Nevertheless, nephrotoxic medication exposure has been recognized as an important contributor to kidney injury after pediatric HCT [16]. In the present study, CKD was significantly more frequent in younger patients. No significant relationship between age and CKD has been consistently demonstrated in pediatric studies.

### Limitations

The principal limitations of this study are its retrospective design, the heterogeneous underlying diagnoses and transplantation characteristics, the limited sample size, and incomplete renal assessments at some follow-up time points. These factors may have reduced statistical power and limit the generalizability of the findings. Another limitation is that the immunosuppressive regimen evaluated in this study included only cyclosporine A; therefore, the potential renal effects of other immunosuppressive agents could not be assessed. Detailed information on the degree of HLA matching, cumulative doses of individual conditioning agents, previous chemotherapy exposure, baseline anemia, and other comorbidities was not consistently available for all patients because of the retrospective study design. In addition, pre-existing differences in renal function among diagnostic groups, particularly the lower baseline GFR observed in patients with Fanconi aplastic anemia, may have introduced confounding when evaluating post-transplant renal outcomes. The lack of systematic assessment of proteinuria and albuminuria represents an additional limitation of the CKD evaluation. Renal follow-up data were not available for all patients at each predefined time point, resulting in different denominators for the month 6 and month 12 CKD analyses. The retrospective design did not allow the reasons for all missing assessments to be determined with certainty. Therefore, potential bias related to incomplete follow-up cannot be excluded and the CKD findings should be interpreted accordingly. Because of the limited sample size and the small number of outcome events within several subgroups, reliable multivariable modeling was not feasible; therefore, the reported associations are based on unadjusted analyses and may be affected by residual confounding.

## 5. Conclusions

Timely and accurate identification of AKI and CKD after HSCT is important for recognizing potentially modifiable factors. In this cohort, AKI occurred in 54.3% of patients and CKD was present in 45.7% of evaluated patients at month 6. None of the patients developed end-stage kidney disease or required dialysis. The heterogeneous patient population and limited follow-up complicate interpretation and comparison of the findings. Longer follow-up and prospective studies with larger pediatric cohorts are needed to clarify determinants of kidney injury after HSCT and optimize management.

## Figures and Tables

**Table 1:** Descriptive characteristics of patients.

		*n*	%
Age (years) [mean ± SD, min–max]		9.1 ± 5.1	0.2–18.8
Gender	Male	34	48.6
	Female	36	51.4
Primary disease	Thalassemia major	28	40.0
	Malignant diseases	15	21.4
	Fanconi aplastic anaemia	12	17.1
	Other	15	21.4
Donor type	Autologous	2	2.9
	Allogeneic Related	61	87.1
	Allogeneic Non-related	7	10.0
Source of stem cell	Bone marrow	45	64.3
	Peripheral	25	35.7
Conditioning regimen	Myeloablative with TBI	9	12.9
	Myeloablative without TBI	42	60.0
	Non-myeloablative	19	27.1

Abbreviations: SD, standard deviation; TBI, total body irradiation.

**Table 2:** Pre-HSCT nephrological evaluations and their distribution by diagnosis [*n* (%)].

		All Patients	Fanconi Aplastic Anaemia	Thalassemia Major	Malignant Diseases	Other
USG	Abnormal	8 (11.4)	6 (50)	2 (7.1)	0 (0)	0 (0)
	Normal	62 (88.6)	6 (50)	26 (92.9)	15 (100)	15 (100)
DMSA	Abnormal	20 (28.6)	9 (75)	3 (10.7)	4 (26.7)	4 (26.7)
	Normal	42 (60.0)	2 (16.7)	21 (75)	11 (73.3)	8 (53.3)
	Not performed	8 (11.4)	1 (8.3)	4 (14.3)	0 (0)	3 (20)
DTPA	Abnormal	3 (4.3)	1 (8.3)	2 (7.1)	0 (0)	0 (0)
	Normal	7 (10.0)	1 (8.3)	3 (10.7)	2 (13.3)	1 (6.7)
	Not performed	60 (85.7)	10 (83.3)	23 (82.1)	13 (86.7)	14 (93.3)
HT	Normal	69 (98.6)	11 (91.7)	28 (100)	15 (100)	15 (100)

Abbreviations: HSCT, hematopoietic stem cell transplantation; USG, ultrasonography; DMSA, dimercaptosuccinic acid; DTPA, diethylenetriaminepentaacetic acid; HT, hypertension.

**Table 3:** Distribution of AKI status of patients in terms of term and diagnosis [*n* (%)].

		First 100 Days	Month 6	Month 12	Month 24
All patients	No AKI	32 (45.7)	62 (91.2)	58 (86.6)	45 (81.8)
	Stage 1	21 (30)	6 (8.8)	9 (13.4)	10 (18.2)
	Stage 2	12 (17.1)	0 (0)	0 (0)	0 (0)
	Stage 3	5 (7.1)	0 (0)	0 (0)	0 (0)
Fanconi aplastic anaemia	No AKI	6 (50)	12 (100)	12 (100)	9 (100)
	Stage 1	3 (25)	0 (0)	0 (0)	0 (0)
	Stage 2	3 (25)	0 (0)	0 (0)	0 (0)
	Stage 3	0 (0)	0 (0)	0 (0)	0 (0)
Thalassemia major	No AKI	16 (57.1)	26 (92.9)	25 (89.3)	20 (87)
	Stage 1	9 (32.1)	2 (7.1)	3 (10.7)	3 (13)
	Stage 2	2 (7.1)	0 (0)	0 (0)	0 (0)
	Stage 3	1 (3.6)	0 (0)	0 (0)	0 (0)
Malignant diseases	No AKI	6 (40)	12 (85.7)	10 (71.4)	6 (54.5)
	Stage 1	3 (20)	2 (14.3)	4 (28.6)	5 (45.5)
	Stage 2	3 (20)	0 (0)	0 (0)	0 (0)
	Stage 3	3 (20)	0 (0)	0 (0)	0 (0)
Other	No AKI	4 (26.7)	12 (85.7)	11 (84.6)	10 (83.3)
	Stage 1	6 (40)	2 (14.3)	2 (15.4)	2 (16.7)
	Stage 2	4 (26.7)	0 (0)	0 (0)	0 (0)
	Stage 3	1 (6.7)	0 (0)	0 (0)	0 (0)

Abbreviations: AKI, acute kidney injury.

**Table 4:** Comparison of patient characteristics in terms of AKI classification of the highest creatinine value within 100 days [*n* (%)].

	No AKI	AKI		
		Stage 1	Stage 2	Stage 3
Age [mean ± SD]	8.6 ± 4.2	8.6 ± 5.7	10.3 ± 5.8	11.7 ± 6.2
Gender				
Male	17 (50)	9 (26.5)	7 (20.6)	1 (2.9)
Female	15 (41.7)	12 (33.3)	5 (13.9)	4 (11.1)
Diagnoses				
Fanconi aplastic anaemia	6 (50)	3 (25)	3 (25)	0 (0)
Thalassemia major	16 (57.1)	9 (32.1)	2 (7.1)	1 (3.6)
Malignant diseases	6 (40)	3 (20)	3 (20)	3 (20)
Other	4 (26.7)	6 (40)	4 (26.7)	1 (6.7)
pre-HSCT USG findings				
Abnormal	5 (62.5)	2 (25)	1 (12.5)	0 (0)
Normal	27 (43.5)	19 (30.6)	11 (17.7)	5 (8.1)
pre-HSCT HT				
Normal	32 (46.4)	20 (29)	12 (17.4)	5 (7.2)
High	0 (0)	1 (100)	0 (0)	0 (0)
Day of the highest creatinine [mean ± SD]	36.6 ± 23.6	44.0 ± 25.3	37.2 ± 25.7	50.2 ± 27.5
post-HSCT HT development				
Yes	20 (62.5)	14 (66.7)	10 (83.3)	3 (60.0)
No	12 (37.5)	7 (33.3)	2 (16.7)	2 (40.0)
Number of days of post-HSCT HT medication use [median (min–max)]	17.5 (5–135)	18 (6–28)	193.5 (22–365)	20 (14–26)
GFR [mean ± SD]				
Basal	104.89 ± 40.14	105.76 ± 41.33	118.46 ± 56.41	105.12 ± 66.23
Discharge	76.29 ± 24.44	77.1 ± 27.71	80.74 ± 38.51	73.22 ± 37.9
Month 3	88.82 ± 74.08	40.2 ± 1.13	37 ±.	53.37 ± 26.26
Month 6	101.69 ± 39.1	91.23 ± 38.79	140.7 ± 72.29	112.43 ± 58.52
Month 12	94.87 ± 31.78	106.6 ± 59.25	114.41 ± 68.59	101.2 ± 49.85

Abbreviations: AKI, acute kidney injury; HSCT, hematopoietic stem cell transplantation; USG, ultrasonography; HT, hypertension; GFR, glomerular filtration rate; SD, standard deviation.

**Table 5:** Comparison of the development of HT after HSCT in patients and related factors [*n* (%)].

	Post HSCT HT Development		*p*
	Yes	No	
Diagnosis			
Fanconi aplastic anaemia	2 (16.7)	10 (83.3)	
Thalassemia major	14 (50.0)	14 (50.0)	
Malignant diseases	1 (6.7)	14 (93.3)	
Other	6 (40.0)	9 (60.0)	
Abnormal USG	3 (13)	5 (10.6)	1.000
Medication use			
Aminoglycoside	5 (21.7)	13 (27.7)	0.809
Amphotericin	2 (8.7)	4 (8.5)	1.000
Teicoplanin	7 (30.4)	14 (29.8)	1.000
Ganciclovir	0 (0)	5 (10.6)	0.164
Voriconazole	0 (0)	3 (6.4)	0.546
Complications			
Acute GVHD	5 (21.7)	5 (10.6)	0.279
Chronic GVHD	0 (0)	3 (6.4)	0.546
VOD	6 (26.1)	6 (12.8)	0.189
Febrile Neutropenia	5 (21.7)	14 (29.8)	0.671
CMV infection	4 (17.4)	8 (17)	1.000
CsA toxicity	3 (6.4)	2 (8.7)	1.000
Treatment regimen			**0.014**
Myeloablative with TBI	0 (0)	9 (100)	
Myeloablative without TBI	19 (45.2)	23 (54.8)	
Nonmyeloablative	4 (21.1)	15 (78.9)	
Creatinine [mean ± SD]			
Basal	0.52 ± 0.14	0.52 ± 0.15	0.992
Highest in 100 days	0.85 ± 0.59	0.97 ± 0.49	0.391
Month 6	0.51 ± 0.11	0.55 ± 0.16	0.205
Month 12	0.49 ± 0.1	0.57 ± 0.13	**0.019**
Month 24	0.51 ± 0.1	0.59 ± 0.16	0.057
GFR [mean ± SD]			
Basal	59.1 ± 28.5	70.2 ± 37.7	0.243
Discharge	37.5 ± 19.2	47.4 ± 15.7	0.081
Month 3	59 ± 43.5	29.4 ± 6.6	0.158
Month 6	48.5 ± 24.5	65.4 ± 24.5	**0.029**
Month 12	49.3 ± 23	61.1 ± 27	0.128

Abbreviations: HSCT, hematopoietic stem cell transplantation; HT, hypertension; USG, ultrasonography; GVHD, graft-versus-host disease; VOD, veno-occlusive disease; CMV, cytomegalovirus; CsA, cyclosporine A; TBI, total body irradiation; GFR, glomerular filtration rate; SD, standard deviation. Statistically significant *p*-values (*p* < 0.05) are indicated in bold.

**Table 6:** CKD status of patients at months 6 and 12 [*n* (%)].

	CKD at Month 6 (*n*:46)	CKD at Month 12 (*n*:56)
Normal	25 (54.3)	31 (55.4)
Stage 1	4 (8.7)	3 (5.4)
Stage 2	7 (15.2)	10 (17.9)
Stage 3	9 (19.6)	11 (19.6)
Stage 4	1 (2.2)	1 (1.8)

Abbreviations: CKD, chronic kidney disease.

**Table 7:** Comparison of age, creatinine and GFR in terms of CKD status of patients at months 6 and 12.

	CKD at Month 6 *n*:46			CKD at Month 12 *n*:56		
	Normal	CKD	*p*	Normal	CKD	*p*
Age [mean ± SD]	11.15 ± 4.67	8.08 ± 4.4	**0.028**	10.34 ± 4.17	7.66 ± 5.04	**0.034**
**Creatinine [mean ± SD]**						
Basal	0.54 ± 0.16	0.53 ± 0.14	0.740	0.53 ± 0.12	0.55 ± 0.19	0.655
Highest in 100 days	0.99 ± 0.69	0.91 ± 0.45	0.635	0.92 ± 0.62	0.94 ± 0.47	0.895
Month 6	0.54 ± 0.12	0.57 ± 0.14	0.447	0.56 ± 0.13	0.52 ± 0.18	0.391
Month 12	0.55 ± 0.12	0.56 ± 0.13	0.771	0.56 ± 0.12	0.54 ± 0.14	0.599
Month 24	0.59 ± 0.17	0.57 ± 0.14	0.696	0.6 ± 0.13	0.56 ± 0.19	0.444
**GFR [mean ± SD]**						
Basal	126.66 ± 46.89	88.16 ± 32.55	**0.005**	116.12 ± 46.35	88.51 ± 37.76	**0.028**
Discharge	84.74 ± 27.89	59.65 ± 19.42	**0.011**	78.73 ± 25.3	63.42 ± 20.12	0.065
Month 3	114.93 ± 65.94	37.72 ± 8.39	0.059	99.82 ± 66.35	36.3 ± 7.44	0.066
Month 6	134.23 ± 34.45	65.98 ± 21.25	**<0.001**	121.81 ± 34.94	70.08 ± 25.55	**<0.001**
Month 12	134.93 ± 45.8	71.95 ± 27.87	**<0.001**	129.08 ± 40.49	62.99 ± 18.76	**<0.001**

Abbreviations: CKD, chronic kidney disease; GFR, glomerular filtration rate; SD, standard deviation. Statistically significant *p*-values (*p* < 0.05) are indicated in bold.

## Data Availability

The data presented in this study are available on reasonable request from the corresponding author due to privacy and ethical restrictions.

## Supplementary Materials

The following supporting information can be downloaded at: https://www.mdpi.com/article/10.3390/jcm15207810/s1. Table S1. Comparison of complication rates and medication use according to the development of AKI in patients [n (%)]. Table S2. Comparison of CKD status at month 6 and 12 according to patient characteristics.
