# Supplementary material for: Renal Complications After Pediatric Hematopoietic Stem Cell Transplantation: A Retrospective Cohort Study
Source: J Clin Med. 2026 Oct 10;15(20):7810. doi: 10.3390/jcm15207810 (PMC13705281; doi:10.3390/jcm15207810)
Supplement: Supplementary file 1 [file jcm-15-07810-s001.zip › jcm-4571390-supplementary.pdf]

Supplementary Materials

Table S1. Comparison of complication rates and medication use according to the development of AKI in patients [n (%)].

	All Patients	AKI			
		No AKI	AKI		
			Stage 1	Stage 2	Stage 3
HT	23 (32.9)	12 (37.5)	7 (33.3)	2 (16.7)	2 (40)
Acute GVHD	10 (14.3)	4 (12.5)	4 (19)	0 (0)	2 (40)
Chronic GVHD	3 (4.3)	2 (6.3)	1 (4.8)	0 (0)	0 (0)
VOD	12 (17.1)	7 (21.9)	4 (19)	1 (8.3)	0 (0)
Febrile Neutropenia	19 (27.1)	7 (21.9)	5 (23.8)	4 (33.3)	3 (60)
CMV infection	12 (17.1)	4 (12.5)	5 (23.8)	2 (16.7)	1 (20)
CsA toxicity	5 (7.1)	0 (0)	3 (14.3)	2 (16.7)	0 (0)
Medication use					
Aminoglycoside	18 (25.7)	8 (25)	4 (19)	3 (25)	3 (60)
Amphotericin	6 (8.6)	4 (12.5)	0 (0)	1 (8.3)	1 (20)
Teicoplanin	21 (30)	10 (31.3)	6 (28.6)	3 (25)	2 (40)
Ganciclovir	5 (7.1)	1 (3.1)	3 (14.3)	1 (8.3)	0 (0)
Voriconazole	3 (4.3)	0 (0)	0 (0)	2 (16.7)	1 (20)
Treatment regimen					
Myeloablative with TBI	9 (12.9)	3 (33.3)	1 (11.1)	3 (33.3)	2 (22.2)
Myeloablative without TBI	42 (60.0)	21 (50)	15 (35.7)	3 (7.1)	3 (7.1)
Nonmyeloablative	19 (27.1)	8 (42.1)	5 (26.3)	6 (31.6)	0 (0)

Abbreviations: AKI, acute kidney injury; HT, hypertension; GVHD, graft-versus-host disease; VOD, veno-occlusive disease; CMV, cytomegalovirus; CsA, cyclosporine A; TBI, total body irradiation.

Table S2. Comparison of CKD status at month 6 and 12 according to patient characteristics.

	CKD at Month 6		CKD at Month 12	
	n46	P	n56	p
**Gender**		0.480		0.721
Male	7 (36.8)		10 (40)	
Female	14 (51.9)		15 (48.4)	
**Diagnosis**		-		-
Fanconi aplastic anaemia	6 (75)		9 (90)	
Thalassemia major	7 (35)		8 (32)	
Malignant diseases	4 (36.4)		4 (28.6)	
Other	4 (57.1)		4 (57.1)	
**AKI status**		-		-
No AKI	10 (43.5)		12 (41.4)	
Stage 1	8 (57.1)		8 (50)	

Stage 2	1 (25)		3 (50)	
Stage 3	2 (40)		2 (40)	
**USG**		0.318		0.121
Abnormal	3 (75)		6 (75)	
Normal	18 (42.9)		19 (39.6)	
**Medication use**				
**Aminoglycoside**		0.740		0.438
No	15 (42.9)		17 (40.5)	
Yes	6 (54.5)		8 (57.1)	
**Amphotericin**		**0.015**		0.161
No	16 (39)		21 (41.2)	
Yes	5 (100)		4 (80)	
**Teicoplanin**		1.000		1.000
No	15 (45.5)		17 (43.6)	
Yes	6 (46.2)		8 (47.1)	
**Ganciclovir**		1.000		0.314
No	20 (45.5)		22 (42.3)	
Yes	1 (50)		3 (75)	
**Voriconazole**		1.000		1.000
No	20 (45.5)		24 (44.4)	
Yes	1 (50)		1 (50)	
**Complications**				
**HT**		0.617		1.000
No	15 (50)		16 (44.4)	
Yes	6 (37.5)		9 (45)	
**Acute GVHD**		0.390		0.272
No	17 (42.5)		19 (40.4)	
Yes	4 (66.7)		6 (66.7)	
**Chronic GVHD**		1.000		0.581
No	20 (45.5)		23 (43.4)	
Yes	1 (50)		2 (66.7)	
**VOD**		1.000		0.312
No	17 (44.7)		22 (48.9)	
Yes	4 (50)		3 (27.3)	
**Febrile neutropenia**		1.000		0.877
No	16 (44.4)		18 (42.9)	
Yes	5 (50)		7 (50)	
**CMV infection**		1.000		0.688
No	18 (46.2)		21 (42.9)	
Yes	3 (42.9)		4 (57.1)	
**CsA toxicity**		0.203		0.620
No	19 (43.2)		24 (46.2)	
Yes	2 (100)		1 (25)	

**Treatment regimen**		-		-
Myeloablative with TBI	3 (42.9)		3 (37.5)	
Myeloablative without TBI	9 (33.3)		10 (29.4)	
Non-myeloablative	9 (75)		12 (85.7)	
**pre-HSCT HT**		0.457		0.446
Normal	20 (44.4)		24 (43.6)	
High	1 (100)		1 (100)	

Abbreviations: CKD, chronic kidney disease; AKI, acute kidney injury; USG, ultrasonography; HT, hypertension; GVHD, graft-versus-host disease; VOD, veno-occlusive disease; CMV, cytomegalovirus; CsA, cyclosporine A; TBI, total body irradiation; HSCT, hematopoietic stem cell transplantation.
